# Cyclophilins as key players in protozoan parasite infections

**DOI:** 10.1186/s13071-025-07098-y

**Published:** 2025-11-11

**Authors:** Reza Mansouri, Enrique Granado-Aparicio, Claudia Alcedo, Julio López-Abán, Reza Shafiei, Antonio Muro, Raúl Manzano-Román, Sajad Rashidi

**Affiliations:** 1 Department of Immunology, Faculty of Medicine, Shahid Sadoughi University of Medical Sciences and Health Services, Yazd, Iran; 2 Infectious and Tropical Diseases Group (E-INTRO), Institute of Biomedical Research of Salamanca-Research Center for Tropical Diseases at the University of Salamanca (IBSAL-CIETUS), Faculty of Pharmacy, University of Salamanca, 37008 Salamanca, Spain; 3 Vector-Borne Diseases Research Center, North Khorasan University of Medical Sciences, Bojnurd, Iran; 4 Molecular Medicine Research Center, Khomein University of Medical Sciences, Khomein, Iran; 5 Department of Basic Sciences, Khomein University of Medical Sciences, Khomein, Iran

**Keywords:** Cyclophilin, Protozoan parasite, Infection, Therapeutic target, Vaccine development

## Abstract

Cyclophilins (Cyps), a highly conserved family of immunophilins with peptidyl-prolyl cis-trans isomerase (PPIase) activity, play pivotal roles in protein folding, cell signaling, immune modulation, and host–pathogen interactions. In protozoan infections, parasite-encoded Cyps are essential for immune evasion, oxidative stress regulation, and pathogen survival. This review highlights the multifaceted roles of Cyps in *Plasmodium*, *Toxoplasma*, *Trypanosoma*, and *Leishmania*, with particular emphasis on their involvement in host cell invasion, replication, and immunomodulation. Key Cyps—such as *Plasmodium* Cyp19B, *Toxoplasma* Cyp18, *Leishmania major* Cyp19, and *Trypanosoma* Cyp19—have been implicated in oxidative stress management, host cell entry, and immune suppression. Given these critical functions, Cyps represent promising therapeutic targets; cyclosporine A (CsA) and its analogs inhibit parasite proliferation by disrupting Cyp activity. Moreover, parasite-derived Cyps show potential as vaccine candidates, eliciting protective immunity in models of leishmaniasis, toxoplasmosis, and Chagas disease. Host Cyps further influence infection outcomes by modulating mitochondrial permeability, cytokine production, and macrophage polarization. For example, mitochondrial CypD regulates cell death pathways in *Trypanosoma cruzi*-induced cardiac pathology. Despite these advances, further research is required to validate the therapeutic and prophylactic potential of Cyps. Future studies should investigate stage-specific Cyp functions, host–pathogen interactions, and the development of nonimmunosuppressive Cyp inhibitors to advance targeted antiparasitic strategies.

**Graphical Abstract:**

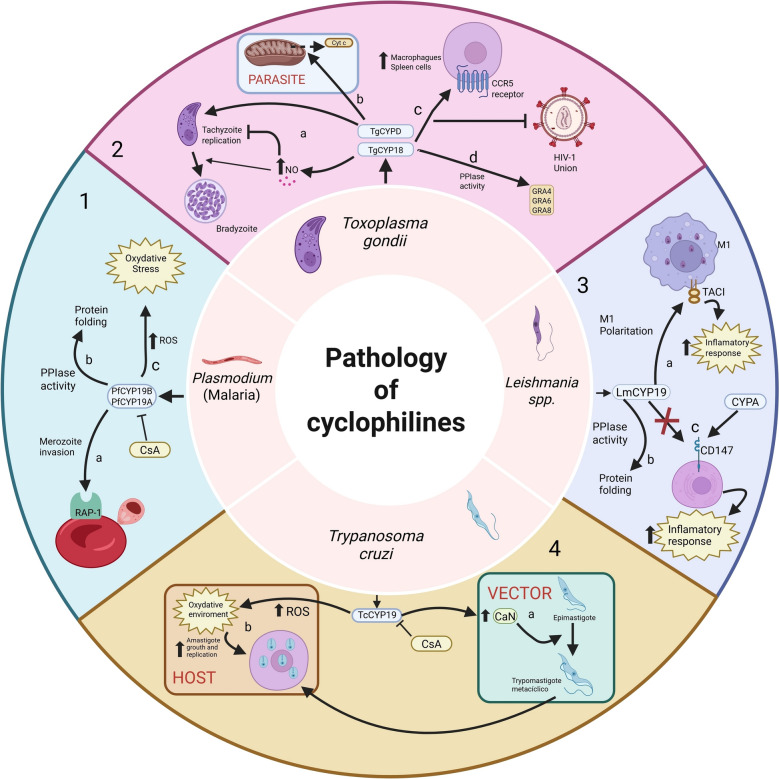

## Background

Immunophilins, a highly conserved family of peptidyl-prolyl isomerases (PPIases), catalyze the cis–trans isomerization of proline peptide bonds, facilitating protein folding and regulating diverse cellular processes [1]. They are classified into two major subfamilies on the basis of their affinity for immunosuppressive drugs: cyclophilins (Cyps), which bind cyclosporine A (CsA), and FK506-binding proteins (FKBPs), which interact with tacrolimus (FK506) and rapamycin [1, 2]. Despite lacking sequence homology, both families perform analogous roles in protein folding and signal transduction [1, 3, 4]. A third, smaller group of parvulin-like PPIases further expands this functional repertoire, operating independently of CsA or FK506 binding and highlighting the structural and mechanistic diversity within the immunophilin family [1].

Structurally, Cyps are characterized by a cyclophilin-like domain (CLD) composed of eight antiparallel *β*-strands flanked by two *α*-helices, whereas FKBPs adopt distinct *β*-sheet–rich folds [5]. Despite these differences, both families are critical for protein trafficking, immune regulation, and stress responses [6]. Genome-wide studies have identified multiple Cyp genes in pathogens and infected host cells, revealing their dual roles in either exacerbating or mitigating disease [7]. For example, CypD acts as a prosurvival signaling molecule, suppressing apoptosis likely through interactions with the adenine nucleotide translocator (ANT), a key component of the mitochondrial permeability transition pore (mPTP) [8]. In contrast, CypA contributes to tissue injury during inflammation and oxidative stress [9, 10].

Emerging evidence also highlights the pathogenic and immunomodulatory roles of extracellular Cyps. In coronavirus-19 (COVID-19), they function as extracellular chemokines, triggering hyperinflammation by sensing severe acute respiratory syndrome coronavirus 2 (SARS-CoV-2) in innate immune cells, a mechanism that positions them as promising therapeutic targets [11, 12]. In addition, both host- and pathogen-derived Cyps are secreted in extracellular vesicles (EVs), where they modulate immune responses and may serve as biomarkers of infection [13].

Given their involvement in viral and bacterial virulence, inflammation, and cancer, Cyp inhibitors are considered compelling therapeutic candidates [4, 14–17]. Their interactions with host and pathogen biomolecules shape infection dynamics and immune regulation, making them essential for understanding disease mechanisms [18, 19]. Consequently, characterizing pathogen-derived Cyps and their signaling pathways could reveal novel treatment and vaccine strategies for infectious and parasitic diseases [9, 10]. This review explores the roles of Cyps in parasitic infections and their potential as therapeutic and prophylactic targets.

### Parasite cyclophilins

**Fig. 1:**
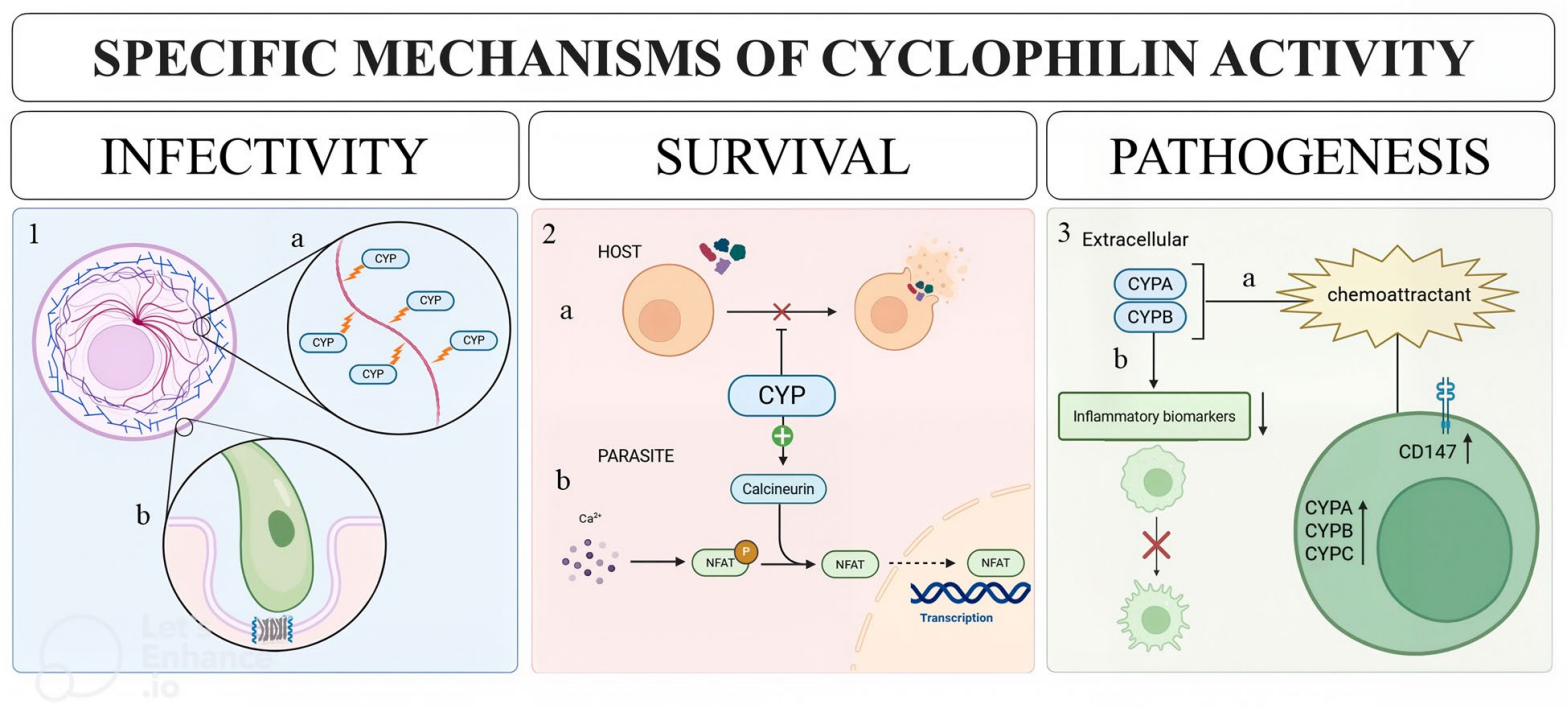
Specific mechanisms of Cyp activity cyclophilins (Cyps) are multifunctional biomolecules involved in various stages of infection. *Early infection*: Cyps interact with host cell structures, facilitating parasite attachment and invasion. (1.a) Cyps hijack the host actin cytoskeleton [18, 81]. (1.b) Formation of Cyp-rich pedestal structures regulates parasite attachment and host cell invasion [18, 82]. *Survival*: Cyps enhance parasite survival by counteracting host defenses and modulating host signaling. (2.a) Neutralization of host lytic defense peptides [23, 83]. (2.b) Activation of parasite calcineurin [23] *Pathogenesis*: Cyps modulate host immune responses and exhibit anti-inflammatory functions. (3.a) Immune modulation: extracellular Cyps (CypA, CypB) chemoattract activated T lymphocytes via CD147 [18, 66, 84]. (3.b) Antiinflammatory activity: suppression of macrophage activation through reduced production of inflammatory biomarkers [85]

Cyclophilins have emerged as critical regulators in diverse protozoan parasites, including intestinal species (*Entamoeba histolytica*, *Giardia intestinalis*, *Eimeria tenella*, *Cryptosporidium parvum*), vaginal species (*Trichomonas vaginalis*), tissue-dwelling species (*Leishmania* spp., *Trypanosoma cruzi*, *Toxoplasma gondii*, *Neospora caninum*), and blood-borne species (*Plasmodium falciparum*, *Babesia bovis*, *Theileria annulata*) (Table 1). These parasite-encoded Cyps orchestrate key cellular processes, including stress adaptation, RNA modulation (e.g., via MED21 and ap65-1), mitochondrial permeability regulation, and reactive oxygen species (ROS) production. Collectively, these mechanisms enhance protozoan replication, host cell invasion, and immune evasion, positioning Cyps as attractive therapeutic targets (Fig. 1).

**Table 1:** Cyclophilins expressed in protozoan parasites

Parasites	Cyps	Biological functions	Cyp ligands and plausible mechanism of action	References
*Entamoeba histolytica*	Cyp (uncharacterized)	Elevated in abscess-derived trophozoites; likely supports replication	CsA inhibits trophozoite replication	[25–27]
*Giardia intestinalis*		Involved in parasite replication		[28]
*Eimeria tenella*	Cyp89-kDa	Promotes parasite replication and host cell invasion	CsA (mechanism uncharacterized)	[18, 29]
*Cryptosporidium parvum*	Cyps (uncharacterized)		CsA and analogs (SDZ 033-243, SDZ PSC-833) inhibit parasite growth	[18, 30]
*Trichomonas vaginalis*	TvCyp1	Binds Myb1, facilitating its nuclear translocation (regulates *ap65-1* gene expression, linked to pathogenesis)	CsA disrupts TvCyp1–Myb1 interaction	[31]
	TvCyp2	Regulates endomembrane trafficking (localizes TvCyp1 and Myb3 in membranes, hydrogenosomes, and plasma membranes)	CsA (mechanism uncharacterized)	[32, 33]
*Leishmania donovani*	Cyp40	Maintains cellular homeostasis under stress; essential for proliferation and viability	CsA (promastigotes: cell cycle arrest, loss of motility; amastigotes: highly toxic)	[34, 35]
	CypA	Critical for parasite survival and persistence	CsA reduces IL-12, TNF-α, IFN-γ and increases IL-10, IL-4, NO, H_2_O_2_; downregulates CypA expression	[36, 37]
			DHCsA-d: inhibits promastigote/amastigote replication, enhances TH1 response (↑IL-12, TNF-α, IFN-γ; ↓IL-10, IL-4, NO, H_2_O_2_); no effect on CypA expression	
*Leishmania major*	Cyp19	Function uncharacterized	CsA binds Cyp19 but lacks calcineurin inhibition; in vivo, enhances IFN-γ and TH1 response	[38]
*Trypanozoma cruzi*	TcCyp19	Modulating ROS production and increasing parasite replication and pathogenesis (involved in drug resistance)	CsA (mechanism uncharacterized)	[39]
	TcCyp21, 25, 28, 34, 40	Uncharacterized	Non-immunosuppressive CsA analogs (H-7–94, F-7–62, MeVal-4) inhibit epimastigote proliferation, trypomastigote penetration, and amastigote development	[40]
	TcCyp22	Regulates parasite cell death	CsA and derivatives (mechanism uncharacterized)	[36]
*Toxoplasma gondii*	TgCyp18	Enhances host cell proliferation and parasite migration	CsA (mechanism uncharacterized)	[41]
	TgCyp23	Uncharacterized	CsA derivatives (NIM811, Alisporivir) inhibit parasite growth	[42]
	TgCyp20, 82,		CsA (mechanism uncharacterized)	[43, 44]
*Neospora caninum*	NcCyp (uncharacterized)	Enhances IFN-γ production and CCR5-dependent cell migration; modulates host-parasite interaction and immune evasion	CsA (mechanism uncharacterized)	[20, 45]
*Plasmodium falciparum*	PfCyp19A, 19B	Mediates parasite invasion		[46–48]
*Theileria annulata*	TaCyp1	Binds host MED21, potentially regulating RNA polymerase II-dependent transcription and cell transformation	Uncharacterized	[49]

*IFN-γ* interferon gamma, *CCR5* CC-chemokine receptor 5, *CsA* cyclosporine A, *DHCsA-d* dihydrocyclosporine A, *ROS* reactive oxygen species, *IL* interleukin, *TNF-α* tumor necrosis factor-alpha, *NO* nitric oxide, *H₂O₂* hydrogen peroxide, *TH1* T-helper 1, *MED21* mediator complex subunit 21, *NIM811/Alisporivir* non-immunosuppressive CsA analogs, *SDZ 033-243/PSC-833* CsA derivatives, *H-7-94/F-7-62/MeVal-4* experimental CsA analogs, *Myb1/Myb3* *Trichomonas vaginalis* transcription factors

Beyond their intracellular roles, secreted Cyps contribute to immune modulation [20, 21]. For example, *T. gondii* and *N. caninum* release Cyps into the extracellular milieu, where they stimulate interferon-γ (IFN-γ) production, a critical component of host defense [20, 21]. Notably, recombinant *N. caninum* Cyp (NcCyp) has demonstrated efficacy as a vaccine candidate, conferring protection in murine models of neosporosis [21].

Similar immunomodulatory functions are observed in helminths, such as *Schistosoma* spp., where Cyps influence host–parasite interactions [22]. Interestingly, some parasite Cyps also counteract insect-derived antimicrobial peptides and activate calcineurin-dependent pathways, further facilitating infection [23]. Given their dual roles in parasite survival and host immune regulation, Cyps represent promising targets for both drug development and vaccine design.

### Cyclophilins expressed in prominent protozoan parasites (*Plasmodium*, *Toxoplasma*, *Trypanosoma*, and *Leishmania*)

Protozoan parasites express a diverse array of peptidyl-prolyl cis/trans isomerases (Cyps), many of which share homology with human orthologs [24]. These enzymes play pivotal roles in protein folding, signal transduction, and virulence, making them valuable targets for understanding parasite biology and developing novel therapeutic or prophylactic interventions [24]. Despite advances in characterizing their genetic and biochemical properties, the stage-specific functions of Cyps throughout parasite life cycles and their contributions to host–pathogen interactions remain poorly understood. This knowledge gap underscores the need for further research to elucidate their mechanistic roles and exploit their potential in drug and vaccine development.

Recent studies have highlighted the utility of specific inhibitory ligands, such as CsA and its analogs (e.g., DHCsA-d, SDZ 033-243, and MeVal-4), in modulating parasite Cyp activity (Table 1). These tools not only facilitate functional studies but also provide a foundation for the development of targeted antiparasitic therapies. By integrating current insights into the biological roles of Cyps in parasites and infected host cells, this review aims to bridge critical gaps in understanding their pathogenicity and therapeutic potential.

### *Plasmodium* spp.

Genomic studies have identified multiple Cyps in *Plasmodium* spp., including PfCyp14, PfCyp19A, PfCyp19B, PfCyp23, PfCyp25, PfCyp26, PfCyp32, PfCyp52, PfCyp72, PfCyp81, and PfCyp87 [50, 51]. These Cyps play critical roles in parasite biology, such as facilitating the export of *P. falciparum* virulence factors and mediating stress responses, including adaptation to heat shock during vector transmission [52, 53]. Notably, some Cyps (e.g., PfCyp72) exhibit host-restricted expression, with no detectable activity in mammalian hosts, highlighting their potential as selective therapeutic targets for malaria intervention [54].

Among these, PfCyp19B has emerged as a key regulator of intra-erythrocytic development. Its expression is significantly reduced in red blood cells (RBCs) containing sickle-trait hemoglobin (HbAS) from patients with malaria, likely owing to the pro-oxidant environment of HbAS-RBCs [55, 56]. Given that HbAS confers protection against severe malaria, this downregulation may impair parasite protein trafficking, particularly the surface display of cytoadherence ligands, potentially attenuating disease severity by limiting vascular adhesion [56].

### *Toxoplasma gondii*

*Toxoplasma gondii* employs sophisticated strategies to ensure successful infection, exploiting both CCR5-dependent and independent pathways. Central to these mechanisms is the secretory protein TgCyp18, which exhibits dual roles in infection dynamics. On one hand, it enhances host defense by stimulating nitric oxide production, suppressing tachyzoite replication. Conversely, TgCyp18 promotes the transition to the chronic bradyzoite stage in a CCR5-dependent manner [57]. Beyond direct parasite regulation, TgCyp18 modulates host immunity by recruiting CD11b^+^ cells to infection sites, involving both CCR5-dependent and independent mechanisms. While TgCyp18 drives macrophage and splenic T lymphocyte proliferation independently of CCR5, it relies on CCR5 to orchestrate cellular migration, thereby expanding the pool of target cells for parasite dissemination [41, 58].

The immunomodulatory effects of TgCyp18 extend to dendritic cells, where it triggers MyD88- and CCR5-dependent signaling to amplify IL-12 production, a cytokine critical for host defense [59–61]. These findings highlight the chaperone-like activity of Cyps and suggest synergistic crosstalk between CCR5 and toll-like receptors to maximize IL-12 responses, skewing innate immunity toward pathways that favor parasite persistence.

Uniquely, TgCyp18 inhibits human immunodeficiency (HIV)-1 cell fusion by selectively targeting CCR5-dependent (R5) viral entry while sparing CXCR4-dependent (X4) strains, a specificity absent in human or *P. falciparum* Cyps [62]. TgCyp18 binds directly to CCR5, blocking syncytium formation between human T cells and effector cells expressing R5 envelopes, without inducing chemokine production or CCR5 downregulation [62]. Competition assays reveal that TgCyp18 interacts with CCR5 at a site overlapping with macrophage inflammatory protein 1 (MIP-1) and HIV-1 R5 gp120 [62], and it suppresses R5 HIV-1 replication in human lymphoid tissues, underscoring its therapeutic potential [63].

Beyond its antiviral role, TgCyp18 localizes to dense granules and the parasitophorous vacuole, where its PPIase activity post-translationally modifies proline-rich parasite proteins (e.g., GRA4, GRA6, GRA8) [44, 64]. These dual functions targeting host CCR5 and remodeling parasite proteins position TgCyp18 as a multifunctional scaffold for novel antiviral strategies.

*Toxoplasma gondii* further exploits Cyp activity through its mitochondrial homolog, TgCypD, which critically regulates tachyzoite invasion and proliferation. *Toxoplasma gondii* CypD depletion suppresses cytochrome c release, enhancing parasite resistance to oxidative stress-induced cell death, and identifies TgCypD as a component of the mPTP, modulating mitochondrial-mediated cell death pathways [65]. Its specialized role in stress adaptation suggests potential targets for intervention.

### *Trypanosoma* spp.

African trypanosomiasis (caused by *T. brucei gambiense* and *T. b. rhodesiense*) and American trypanosomiasis (Chagas disease, caused by *T. cruzi*) are distinct infections with unique pathophysiology. In *T. cruzi*, the secreted PPIase TcCyp19 (19 kDa) plays multifaceted roles in host–parasite interactions [66]. This protein is expressed across all parasite life stages, epimastigotes, trypomastigotes, and amastigotes, and is secreted into the host cytosol during infection [36, 66]. *Trypanosoma cruzi* Cyp19 shares structural and functional homology with Cyps in other trypanosomatids, including *L. major* (LmCyp19) and *T. brucei* (TbCyp19), suggesting evolutionarily conserved roles [67].

A key function of TcCyp19 is activating calcineurin-mediated signaling, driving partial transformation into metacyclic trypomastigotes, a crucial step for host cell invasion [66]. Cyclophilin inhibitors (e.g., cyclosporine A) significantly reduce parasite proliferation, underscoring TcCyp19’s contribution to infectivity [68]. Within the insect vector, epimastigote-secreted TcCyp19 neutralizes antimicrobial peptides, enhancing parasite survival in both mammalian and insect hosts [23].

*Trypanosoma cruzi* Cyp19 induces ROS production in host cells via NADPH oxidase (NOX2), creating an environment favorable for intracellular amastigote replication [23, 66, 69]. CRISPR/Cas9 studies show that TcCyp19-deficient parasites have impaired growth due to diminished ROS, a phenotype reversible upon re-expression [66]. Notably, TcCyp19 is overexpressed in benznidazole-resistant strains, implicating it in drug resistance [67]. Its secretion also triggers host antibody responses, making it a potential biomarker for therapeutic efficacy [68].

Despite progress, key knowledge gaps remain. The extent to which TcCyp19 mimics mammalian CypA, such as binding to CD147 to activate ERK1/2 signaling, warrants further investigation [66]. Its role in chronic Chagas disease manifestations, particularly cardiac inflammation, remains to be fully elucidated, although parallels with CypA-driven autoimmune pathways are emerging [70–72]. Addressing these gaps may reveal novel therapeutic targets for the management of Chagas disease.

Cyclophilins have been extensively studied in *Leishmania* spp. In particular, *L. donovani* CypA (LdCypA) has been characterized in terms of thermal stability, crystal structure, and aggregation behavior, while *L. infantum* isolates from canine hosts express LiCyp2 and LiCyp40, implicating Cyps in stress response and intracellular survival [35, 73–75]. Functional studies suggest that LdCyp40 contributes to stress homeostasis, potentially supporting parasite persistence during infection [75].

The interplay between *Leishmania* Cyps and host immune mechanisms reveals evolutionary adaptations that facilitate parasite survival. In *L. major*, the predominant isoform LmCyp19 retains enzymatic activity but diverges functionally from human cyclophilin A (hCypA) [76]. Structural analyses show that while LmCyp19 preserves the CsA-binding site, it lacks heparan-binding motifs required for interaction with CD147, a host receptor critical for inflammatory signaling [76–78]. This divergence has key immunological consequences: whereas hCypA–CD147 interactions trigger the activation of immune cells (e.g., neutrophils and macrophages), *L. major* exploits LmCyp19’s inability to bind CD147 to evade early immune detection [76, 78]. Experimental evidence confirms that restoring heparan-binding motifs in LmCyp19 reinstates CD147-dependent signaling, highlighting *Leishmania*’s adaptation to silence this pathway [76]. Supporting this, CsA, a CypA inhibitor, fails to impair *Leishmania* infectivity in macrophages, underscoring the parasite’s exploitation of Cyp functional differences to circumvent host defenses [77]. Critical gaps remain in understanding species-specific variations in Cyp-mediated immune modulation. For instance, LdCyp shares structural conservation with hCypA but exhibits resistance to CsA, suggesting species-specific adaptations. Future studies should investigate whether Cyp polymorphisms correlate with disease severity or therapeutic outcomes, particularly in the context of emerging drug resistance. [77, 79].

**Fig. 2:**
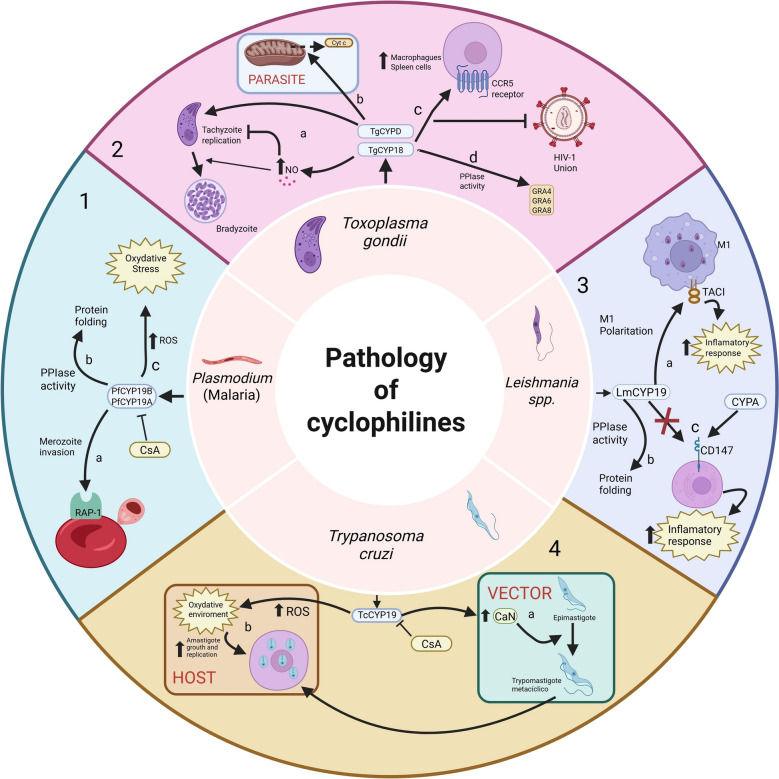
Targets and functions of parasite Cyps. **PfCyp19B and PfCyp19A in *P. falciparum****.* (1.a) Facilitate merozoite invasion. (1.b) Exhibit PPIase activity, ensuring proper folding of parasite proteins [51]. (1.c) Participate in the *Plasmodium* reactive oxidative stress complex [86]. **TgCyp18 and TgCypD in *T. gondii****.* (2.a) TgCyp18 inhibits tachyzoite growth and promotes tachyzoite-to-bradyzoite transition, whereas TgCypD enhances tachyzoite growth [57]. (2.b) TgCypD regulates cytochrome c release from parasite mitochondria. (2.c) TgCyp18 interacts with the CCR5 receptor, attracting immune cells and blocking HIV-1 entry [62]. (2.d) PPIase activity facilitates proper protein folding. **LmCyp19 in *Leishmania* spp.** (3.a) Activates the TACI receptor, polarizing macrophages toward an M1 phenotype and amplifying inflammatory responses [87]. (3.b) Exhibits PPIase activity to aid protein folding [77]. (3.c) Unlike other Cyps, it does not interact with CD147 [76]. (4) **TcCyp19 in *T. cruzi****.* (4.a) Promotes the epimastigote-to-trypomastigote transition in the insect vector [66]. (4.b) Generates an oxidative environment conducive to amastigote growth and replication in the host [66]

Beyond immune evasion, Cyps play a crucial role in parasite metabolism. The interaction between LdCyp and adenosine kinase (AdK) is essential for metabolic homeostasis, particularly in modulating purine salvage pathways critical for parasite proliferation [80]. Structural and mutational analyses have identified key residues in LdCyp’s catalytic and binding domains that mediate this interaction. Disrupting these residues impairs binding efficiency and reduces parasite viability in vitro, positioning the LdCyp–AdK axis as a potential therapeutic target [80]. Further exploration of the structural dynamics of this interaction could inform the development of novel antileishmanial drugs. A summary of key targets and functions of parasite Cyps is provided in Fig. 2.

### Cyclophilins expressed in host cells

Emerging evidence underscores the critical role of host cyclophilins (hCyps) in modulating immune responses and cellular pathways during parasitic infections. For instance, upregulation of CypA in coccidia-infected host cells suggests its involvement in host defense mechanisms [88], while *Haemaphysalis longicornis*-derived CypA has been implicated in immune regulation during *Babesia* infections [89].

A striking example is mitochondrial CypD, encoded by the *Ppif* gene, which plays a pivotal role in *T. cruzi* infection. *Ppif*^-^/^-^ mice exhibit significantly reduced parasite loads in cardiac and skeletal tissues, along with diminished infection rates in cardiomyocytes and macrophages [90]. These findings indicate that CypD inhibition stabilizes mitochondrial membrane potential, thereby attenuating *T. cruzi* pathogenicity and dissemination [90], highlighting CypD as a promising therapeutic target to mitigate cardiac damage in Chagas disease.

While conventional antiparasitic drugs primarily target pathogen-encoded proteins, recent therapeutic strategies have shifted toward host cell pathways essential for parasite survival. For example, CsA-mediated inhibition of host CypA impairs *L. major* replication in macrophages, emphasizing the potential of host-directed therapies [91, 92]. Although the precise mechanism remains unclear, it may parallel CsA’s ability to block HIV-1 replication by interfering with host Cyp interactions [89, 93]. Supporting this concept, antisense oligonucleotides targeting host CypA in infected macrophages reduce amastigote replication, reinforcing Cyps as critical facilitators of parasitic survival [91].

Beyond parasite-induced modulation, several human Cyps, including hCypA, hCypB, hCypD, and hCyp40, exhibit structural homology with parasite Cyps, implicating them in infection dynamics (Table 2). Notably, hCypA facilitates *L. major* infection and shares homology with protozoan Cyps such as PfCyp19 (*P. falciparum*), TgCyp18 (*T. gondii*), and TcCyp19 (*T. cruzi*) [36]. Similarly, hCypB participates in *P. falciparum* merozoite invasion [94], while hCypD regulates mitochondrial membrane permeability, inhibiting apoptosis and promoting *T. cruzi* infection [90]. Although hCyp40 has not been directly linked to protozoan infections, its structural resemblance to TcCyp40 (*T. cruzi*) and LdCyp40 (*L. donovani*) suggests potential involvement [35]. Given these interactions, hCyps represent plausible targets for immunosuppressive drugs such as CsA (Table 2).

**Table 2:** Human cyclophilins (hCyps): biological functions and roles in parasite infections

hCyps	Biological function	Interaction in parasite infections	References
hCypA	Upregulates cytokines/chemokines (CXCL2, CXCL3, CXCL8, IL-1α, IL-1β) Binds CD147 for chemotaxis Modulates RIG-I-mediated antiviral response Activates MMP-2/MMP-9 and promotes VSMC migration Induces endothelial cell apoptosis Enhances VCAM-1/ICAM-1 expression Activates inflammatory pathways	Enhances *L. major* infection Structural analogue of PfCyp19, TgCyp18, LmCyp19, and TcCyp19 Sensitive to CsA	[36, 94, 97]
hCypB	Chaperone for collagen folding Triggers DNA degradation in TCR-stimulated thymocytes Mediates chemotaxis and integrin-dependent adhesion in memory CD4^+^ T cells	Receptor for PfRhopH3 during *P. falciparum* invasion Sensitive to CsA	[94, 98]
hCypC	Associated with macrophage activation Elevated in ischemia and CAD	No known parasite interactions	[99]
hCypD	Regulates mPTP formation and apoptosis Modulates mitochondrial energy metabolism via CypD-OSCP interaction Promotes tumor progression	Inhibits mitochondrial membrane collapse, facilitating *T. cruzi* infection TgCypD functions as a homologue Sensitive to CsA	[90–101]
hCypE	Regulates osteoblast differentiation	No known parasite interactions	[102]
hCyp40	Component of inactive steroid receptor complexes	-TcCyp40 and LdCyp40 act as homologues Sensitive to CsA	[35, 100]

*CAD* Coronary artery disease, *CD147* Cluster of Differentiation 147 (extracellular matrix metalloproteinase inducer), *CsA* Cyclosporine A, *CXCL* CXC motif chemokine ligand (e.g., CXCL2, CXCL3, CXCL8), *hCyp* Human cyclophilin, *ICAM-1* Intercellular Adhesion Molecule 1, *IL-1α/β* Interleukin-1 alpha/beta, *MMP-2/MMP-9* Matrix metalloproteinase-2/-9, *mPTP* Mitochondrial permeability transition pore, *OSCP* Oligomycin sensitivity conferring protein, *PfRhopH3* *Plasmodium falciparum* rhoptry protein H3, *RIG-I* Retinoic acid-inducible gene I (innate immune sensor), *TCR* T-cell receptor, *VSMC* Vascular smooth muscle cell, *VCAM-1* Vascular cell adhesion protein 1

Collectively, these findings highlight the therapeutic potential of targeting host Cyps to combat intracellular parasites while mitigating drug resistance and host toxicity [95, 96]. Beyond parasite-encoded Cyps, identifying host Cyps modulated during infection could yield novel insights into parasite–host interactions, pathogenesis, and therapeutic strategies.

### Therapeutic strategies with Cyp-relevant compounds

The ability to simultaneously target multiple pathophysiological pathways underscores the therapeutic potential of cyclophilin inhibitors (CypIs) [103]. Among these, cyclosporine A (CsA) and its derivatives exhibit high-affinity, selective inhibition of Cyps, with nanomolar binding and potent immunosuppressive effects [104]. Mechanistically, CsA forms a stable complex with Cyp, suppressing its PPIase activity and inhibiting the calcium-dependent phosphatase calcineurin. This prevents nuclear translocation and T-cell activation, a critical step in adaptive immunity [105, 106]. While CsA effectively inhibits Cyp-dependent PPIase activity, its immunosuppressive effects via the Cyp–CsA–calcineurin axis may limit broader applicability, motivating the development of CypIs with improved target specificity and reduced side effects [107].

Cyclosporine A shows broad-spectrum antiparasitic activity by targeting both parasite-encoded and host-derived Cyps [34]. It interferes with critical pathways in *Plasmodium* spp. (*P. berghei*, *P. chabaudi*, *P. falciparum*), *T. cruzi*, *T. gondii*, *Eimeria* spp. (*E. tenella*, *E. mitis*, *E. vermiformis*), and *C. parvum* (Table 1) [54, 108]. Adjunctive therapy combining CsA or related compounds with conventional antiparasitic drugs could enhance efficacy through synergistic mechanisms.

Nonimmunosuppressive CypIs such as alisporivir, SCY-635, NIM811, and CRV431 have shown promising antimalarial activity by selectively inhibiting *P. falciparum* intraerythrocytic proliferation [109]. *Plasmodium falciparum* Cyp19A and PfCyp19B are the principal CsA-binding proteins in *P. falciparum*, with PfCyp19B upregulated during the schizont phase [110]. *Plasmodium falciparum* Cyp19B is overexpressed in artemisinin and piperaquine-resistant strains, contributing to partial drug resistance via modulation of the integrated stress response (ISR) through eIF2α phosphorylation [55, 111]. Alisporivir, with potent inhibitory effects on ring-stage *P. falciparum* and a favorable safety profile, represents a promising monotherapy or combination therapy [112].

Host-derived Cyps also contribute to parasite invasion. Cyclophilin B (CypB) interacts with the merozoite surface protein PfRhopH3 in *P. falciparum*, facilitating erythrocyte invasion. Pharmacological inhibition using CsA or CDP3 peptide impairs invasion, highlighting CypB’s functional relevance, although some studies suggest the PfRhopH3-CypB interaction may be dispensable [113, 114]. Polymorphisms or expression changes in Pfmdr1 (encoding Pgh1) influence *P. falciparum* susceptibility to CsA, potentially by disrupting metabolite transport or chemical gradients in the digestive vacuole [115, 116].

In *T. cruzi*, 15 paralogous Cyps have been identified, with TcCyp19, TcCyp22, TcCyp28, and TcCyp40 experimentally validated as CsA-binding proteins [36, 108, 117]. In *L. major*, CsA resistance correlates with an arginine-to-asparagine substitution in LmCyp19, disrupting complex formation with calcineurin [38]. In addition, phosphorylation of Cyp40 during promastigote-to-amastigote differentiation may regulate virulence and downstream signaling [118–120].

### Cyclophilin-based vaccines in protozoan parasites

Parasitic Cyps play pivotal roles in host immune modulation, influencing T-cell responses, cytokine profiles, and parasite adaptation, making them attractive vaccine candidates [84].

- *Leishmania*: *Leishmania infantum* Cyp1 (LiCyp1) is expressed in both amastigotes and promastigotes. Immunization of BALB/c mice with recombinant LiCyp1 induced long-lasting, partially protective immunity, reducing parasite burden in the liver and spleen. Protection correlated with expansion of antigen-specific CD4^+^ and CD8^+^ memory T cells [121], highlighting LiCyp1 as a promising vaccine candidate.
- *Toxoplasma*: recombinant BCG expressing TgCyp (rBCG-TgCyp) elicited robust humoral and cellular immunity in BALB/c mice following challenge with the virulent *T. gondii* RH strain, with expansion of CD4^+^/CD8^+^ T cells and increased Th1 cytokines (IFN-γ, IL-2, IL-12) [122]. Similarly, a DNA vaccine encoding TgCyp (pVAX1-TgCyp) induced antigen-specific cellular immunity and nitric oxide-mediated tachyzoite suppression, though it only moderately extended survival and did not significantly reduce cerebral parasite burden [123].
- *Trypanosoma*: *Trypanosoma cruzi* Cyp19, secreted throughout the *T. cruzi* life cycle, facilitates intracellular proliferation via ROS generation and suppresses insect antiparasitic peptides [66, 124]. Mice immunized with TcCyp19-deficient parasites developed robust Th1 responses and parasite-specific trypanolytic antibodies, achieving complete protection against acute infection [39, 70], supporting TcCyp19 as a candidate for subunit or live-attenuated vaccines.
- *Neospora*: recombinant NcCyp (19.4 kDa, 86% homologous to TgCyp18) elicits Th1-polarized immunity, with strong IFN-γ secretion and activation of CD4^+^ T cells in bovine peripheral blood mononuclear cells (PBMCs). Vaccination with NcCyp and NcProfilin partially protected sheep against abortion and transplacental transmission [125]. *Neospora caninum* Cyp combined with oligomannose-coated liposomes (OML) activated NF-κB and IL-12p40 secretion in macrophages, inducing robust antibody and cellular immunity in BALB/c mice, providing protection against lethal challenge [126]. These studies underscore TLR2 signaling as critical for NcCyp-OML-induced immunity, highlighting its potential as a neosporosis vaccine candidate.

## Discussion

Cyclophilins play multifaceted roles in protozoan parasite infections, serving as critical mediators of host–pathogen interactions, immune evasion, and parasite survival (Table 1). Despite their functional importance, the precise mechanisms underlying these roles remain partially elucidated. The data reviewed here indicate that: (1) Cyps are evolutionarily conserved across *Plasmodium*, *Toxoplasma*, *Trypanosoma*, and *Leishmania*, exhibiting versatile, genus-specific functions; (2) their roles are often stage-dependent, influencing pathogenesis at distinct life-cycle stages; and (3) their dual potential as targets for drug development and vaccine candidates warrants further exploration.

The structural and functional conservation of Cyps across protozoan parasites underscores their evolutionary significance. For instance, *Plasmodium* PfCyp19B facilitates export of surface proteins such as PfEMP1, critical for erythrocyte invasion [56], while *T. gondii* TgCyp18 modulates host CCR5-dependent signaling to promote bradyzoite differentiation [57, 62]. Cyclophilins often exhibit dual roles: *T. cruzi* TcCyp19 enhances oxidative stress to support amastigote replication [39], whereas *Leishmania* LmCyp19 evades CD147-mediated immune detection [76]. Such functional divergence suggests that Cyps are finely tuned to niche-specific adaptations, making them attractive targets for intervention.

Cyclosporine A and its analogs (e.g., alisporivir, NIM811) inhibit parasite Cyps, though immunosuppressive effects limit clinical applicability [105, 109]. Stage-specific efficacy, for example, PfCyp19B in schizonts [110] and synergy with antimalarials [127], suggests opportunities for optimized dosing or combination therapies. However, Cyp isoform diversity complicates drug design: *Plasmodium* encodes 12 Cyps, with PfCyp19A/B CsA-sensitive, while PfCyp23 is resistant [42, 50]. Similarly, in *T. cruzi*, TcCyp19 and TcCyp22 bind CsA, whereas TcCyp18.4 shows low affinity [40]. Nonimmunosuppressive, selective Cyp inhibitors remain a priority, including elucidating mechanisms such as PfCyp19B-mediated artemisinin resistance via redox regulation or protein folding [111].

Host Cyps contribute to pathogenesis, offering avenues for host-directed therapies (HDT) [128]. *T. cruzi* induces cardiac damage via mitochondrial CypD [90], while *Leishmania* exploits host CypA to sustain M2 macrophage polarization [91]. Targeting these pathways with tissue-specific strategies, such as nanocarrier-delivered siRNA against CypA or the CypD inhibitor sanglifehrin A, may mitigate pathology without systemic immunosuppression [90, 91]. Supporting evidence includes antisense oligonucleotides against macrophage CypA reducing *Leishmania* replication [91] and *Ppif − / − *mice (lacking CypD) exhibiting reduced cardiomyocyte damage during *T. cruzi* infection [90]. Repurposing nonimmunosuppressive Cyp modulators could thus offer therapeutic potential for Chagas cardiomyopathy.

Challenges remain, including stage-specific Cyp functions, host–pathogen interactions, and resistance mechanisms. Cyp isoform expression is dynamically regulated during parasite differentiation, and pharmacological agents may alter transcription [25, 129, 130]. The stage-specific efficacy of Cyp inhibitors, such as CsA, reflects divergent roles across the parasite life cycle [34]. Resistance-associated changes, such as Pfmdr1 overexpression in *P. falciparum* or reduced CypA in antimony-resistant *Leishmania* [131], highlight the need for stage-specific, resistance-aware approaches.

Protozoan Cyps elicit protective immune responses in animal models, although efficacy depends on the parasite strain, adjuvant, and delivery system. Recombinant *L. infantum* LiCyp1 induces memory T-cell responses [121], while DNA vaccines (pVAX1-TgCyp) skew immunity toward Th1 [123]. *Neospora caninum* Cyp combined with oligomannose liposomes activates TLR2-dependent IFN-γ production [126], demonstrating adjuvant-enhanced immunogenicity. Challenges remain as transiently expressed Cyps (e.g., PfCyp19B in schizonts) [126] necessitate multivalent vaccine designs, while immunosuppressive Cyps (e.g., LmCyp19) may require epitope modification or truncation [76]. Translational gaps exist, as most studies rely on murine models; large-animal trials (e.g., sheep for *Neospora*) [125] are needed to evaluate clinical potential.

Emerging evidence implicates Cyps in helminth infections, including *Echinococcus granulosus*, with its allergenic properties [132, 133], and *Schistosoma* spp. immunomodulatory roles [84, 85], positioning Cyps as pan-parasitic targets for novel interventions.

## Conclusions

Cyclophilins serve as a critical interface between parasite survival and host defense, making them attractive targets for therapeutic intervention and vaccine development. Their structural diversity and multifunctional roles, from immune modulation to pathogenesis, underscore their translational potential [134]. While challenges remain, including CsA-associated immunosuppression and variable vaccine efficacy, advances in structural biology and adjuvant systems offer promising pathways for translation.

Key priorities for future research include: (i) elucidating stage-specific Cyp functions, including intracellular/extracellular localization and release mechanisms, to understand dual roles in inflammation and pathology, (ii) developing selective inhibitors targeting parasite-specific Cyps, (iii) exploiting host-directed immunomodulation strategies, (iv) modulating host Cyp PPIase activity via signaling pathways such as PI3K–Akt–mTOR [135, 136]; (v) designing dual-target inhibitors (e.g., hybrid molecules disrupting both parasite Cyps and host inflammatory pathways), and (vi) evaluating Cyp expression dynamics following antiparasitic treatment to uncover molecular mechanisms [137, 138].

A deeper understanding of parasite Cyp–host interactions is essential. Structural biology and gene-editing tools (e.g., CRISPR-Cas9) can elucidate how parasite Cyps manipulate host pathways such as epigenetic regulation, autophagy, or nuclear factor (NF)-κB signaling [59, 126]. High-resolution techniques (Cryo-EM, x-ray crystallography) and omics approaches (single-cell transcriptomics, proteomic mapping of host Cyp networks) may identify stage-specific targets and host–pathogen interfaces.

Nonimmunosuppressive Cyp analogs and peptide mimetics are promising alternatives to CsA [34, 54]. Unique parasite features, such as TgCyp18’s CCR5-binding motif or LmCyp19’s avoidance of CD147, enable selective inhibition [5, 57, 62, 76]. Combination therapies with immunomodulators (e.g., checkpoint inhibitors) could enhance efficacy.

Secretory Cyps remain compelling vaccine candidates. Multi-epitope vaccines, combining Cyps with antigens such as Leishmania HASPB, may improve immunogenicity [139, 140]. Prime-boost strategies (e.g., DNA vaccine + protein-adjuvant boost) and identification of protective peptide loops could refine vaccine design and diagnostic applications [123, 141].

## Data Availability

Data supporting the main conclusions of this study are included in the manuscript.
